# Experiences from 4 Years of Organization of an External Quality Assessment for Mycobacterium tuberculosis Whole-Genome Sequencing in the European Union/European Economic Area

**DOI:** 10.1128/spectrum.02244-22

**Published:** 2022-12-08

**Authors:** R. M. Anthony, E. Tagliani, V. Nikolayevskyy, R. de Zwaan, A. Mulder, M. Kamst, C. Ködmön, M. J. van der Werf, D. Cirillo, D. van Soolingen, ERLTB-Net members

**Affiliations:** a National Tuberculosis Reference Laboratory, Centre for Infectious Disease Control, National Institute for Public Health and the Environment, Bilthoven, The Netherlands; b Emerging Bacterial Pathogens Unit, Division of Immunology, Transplantation and Infectious Diseases, IRCCS San Raffaele Scientific Institute, Milan, Italy; c Department of Infectious Diseases, Imperial College London, London, United Kingdom; d European Centre for Disease Prevention and Control, Stockholm, Sweden; National Mycobacterium Reference Service South, London, UK, and North and Central, Birmingham, UK; Alexander Indra, AGES—Institute for Medical Microbiology and Hygiene, Department for Mycobacteriology and Clinical Molecular Biology, Vienna, Austria; Reference Laboratory Tuberculosis and Mycobacteria Brussels, Belgium; International Reference Laboratory of Mycobacteriology, Statens Serum Institut, Copenhagen, Denmark; Mycobacterial Reference Laboratory, Expert Microbiology Unit, Finnish Institute for Health and Welfare, Helsinki, Finland.; National Reference Centre for Mycobacteria, Pitié-Salpêtrière Hospital, APHP, Sorbonne Université, France; Research Center Borstel, Leibniz Lung Center, Borstel, Germany; Irish Mycobacteria Reference Laboratory, St James’s Hospital, Dublin/Department of Clinical Microbiology, School of Medicine, University of Dublin, Trinity College, Dublin, Ireland; Emerging Bacterial Pathogens Unit, Division of Immunology, Transplantation and Infectious Diseases, IRCCS San Raffaele Scientific Institute, Milan, Italy; Service MycoBac, Laboratoire National de Santé, Dudelange, Luxembourg; Division of Infection Control, Norwegian Institute of Public Health, Oslo, Norway; National Reference Laboratory for Mycobacteria, National Institute of Health “Dr. Ricardo Jorge,” Lisbon, Portugal; National Reference Laboratory for Mycobacterial, National Centre for Microbiology, Instituto de Salud Carlos III, Madrid, Spain; Laboratorio de Investigación Molecular, Instituto Aragonés de Ciencias de la Salud, CIBER Enfermedades Respiratorias, Zaragoza, Spain; National Reference Laboratory for Tuberculosis, Public Health Agency of Sweden, Solna, Sweden; Taichung Veterans General Hospital

**Keywords:** *Mycobacterium tuberculosis*, *Mycobacterium*, genome analysis, quality assurance

## Abstract

Here, we report the development and key features of the first external quality assessment (EQA) scheme for Mycobacterium tuberculosis whole-genome sequencing (WGS). The results of four rounds (2017 to 2020) of implementation within the European tuberculosis reference laboratories network (ERLTB-Net-2) are presented and discussed. EQA panels comprising 10 genomic DNAs were distributed to ERLTB-Net 2 laboratories volunteering to participate in this exercise. Since 2018, five FASTQ files were added to better assess the dry WGS processes, and in 2020, three of the five files were replaced by synthetic files (providing additional flexibility for the mutations included in the panels). Ten National tuberculosis reference laboratories participated in all four EQA rounds, and seven participated in at least one. High-confidence resistance mutations were correctly identified by all laboratories, but challenges remained with respect to the identification of mixed loci and interpretation of rare mutations. M. tuberculosis genotyping and clustering analysis was >90% accurate for pure samples with the main challenges being related to the analysis of mixed genotypes and DNA FASTQ files. The development and implementation of this WGS EQA scheme has contributed to the continuous improvement in performance of participating laboratories in M. tuberculosis WGS and data analysis. This scheme can serve as a model of comprehensive quality assessment for M. tuberculosis WGS that can be replicated in different settings worldwide.

**IMPORTANCE** The wider availability of whole-genome sequencing (WGS) coupled to new developments in bioinformatic tools and databases to interpret Mycobacterium tuberculosis complex WGS data has accelerated the adoption of this method for the routine prediction of antimycobacterial drug resistance and genotyping, thus necessitating the establishment of a comprehensive external quality control system. Here, we report 4 years of development and results from such a panel.

## INTRODUCTION

A dramatic decline in the cost of whole-genome sequencing (WGS) and availability of WGS infrastructure, along with analysis methods for Mycobacterium tuberculosis sequence data (1–6), has resulted in WGS being implemented by many tuberculosis (TB) national reference laboratories (NRLs) in the European Union/European Economic Area (EU/EEA). WGS can be used for both resistance detection and epidemiological typing/(sub)species identification, meaning that WGS, in addition to being more accurate, is also potentially more cost efficient than previous approaches (7). WGS results generally become available before (full) culture-based susceptibility testing results, and the increased typing resolution allows more accurately targeted epidemiological investigations (8, 9). For these reasons, WGS results are increasingly used to support patient treatment and for public health interventions. M. tuberculosis WGS data generation and analysis involves many (nonstandardized) steps and complex interpretation (2, 10); thus, quality control is particularly important. Development of international EQA schemes would also support M. tuberculosis WGS standardization and interpretation of the results in the international context, thus contributing to the harmonization of drug resistance prediction (11) and the quality of epidemiological investigations and supporting the potential of WGS to detect uncommon forms of resistance, which are only rarely routinely encountered by any individual laboratory. Furthermore, participation in an EQA scheme is a requirement for ISO 15189 accreditation and contributes to maintaining and improving laboratory proficiency.

The European Reference Laboratory Network for TB (ERLTB-Net-2), comprising 31 TB NRLs in the European Union/European Economic Area member states and the United Kingdom, was established with the aim of consolidating and strengthening TB laboratory capacity, improving quality, and achieving sustainability in TB laboratory diagnosis through provision of training, harmonization of laboratory methods, and development and implementation of reliable EQA systems and standards in TB laboratory diagnosis within the EU/EEA and beyond, as well as supporting the functionality of national TB laboratory networks.

One of the major network activities is related to the development of a sustainable external quality assurance (EQA) system. Since 2010, the ERLTB-Net has developed and implemented several EQA schemes covering all TB laboratory diagnostic methods, from microscopy to M. tuberculosis identification using rapid molecular diagnostics, culture, genotypic- and phenotypic-based antimicrobial susceptibility testing, and molecular typing (i.e., mycobacterial interspersed repetitive units variable number of tandem repeats [MIRU-VNTR]) (8, 12–14).

With the gradual shifting from MIRU-VNTR to WGS for M. tuberculosis genotyping in several EU/EEA member states and with its increased use for M. tuberculosis drug resistance prediction, the ERLTB-Net-2 coordination team decided in 2016 to develop and implement a specific EQA scheme to assess the EU laboratory proficiency for detecting drug resistance and perform M. tuberculosis relatedness analysis.

To our knowledge, this is the first EQA scheme for M. tuberculosis WGS being developed and implemented on a large scale. Here, we describe its development and report the results of the 4-year (2017 to 2020) implementation within ERLTB-Net-2.

## RESULTS

In 2017, 2018, 2019, and 2020, a total of 13, 12, 14, and 15 EU/EEA TB NRLs of 32, participated in the EQA exercise, respectively.

### Sequencing technology used.

All laboratories utilized Illumina (San Diego, CA, USA) technology to generate their sequence data, apart from one laboratory that used IonTorrent (Thermo Fisher Scientific, USA) for all four EQA rounds.

### Time line for result submission.

After the samples were shipped to participating laboratories, the results were expected within 6 weeks. In 2017, only 5 (38.5%) of the 13 laboratories that joined the EQA activity returned the results within the deadline; the remaining labs requested extensions of up to 21 days. In 2018, only three (25%) laboratories requested an extension. In 2019, two (14.3%) laboratories requested a short extension and returned the results within 8 days of the deadline. In 2020, the great majority, 14 of 15 laboratories, met the deadline, while one laboratory requested an extension due to a COVID-related delay in their sequencing provider.

### Scores.

In 2017, 12 (92.3%) of 13 laboratories scored >75 points and received the EQA proficiency certificate. The remaining laboratory missed the identification of the multidrug-resistant tuberculosis (MDR-TB) isolate due to an error in the analysis pipeline, thus scoring <75 points. In 2018, the number of laboratories receiving the proficiency certificate went down to 10 (83.3%) of 12, and in 2019 and 2020, 100% of participating laboratories received the proficiency certificate.

### Detection and reporting of resistance-associated genotypes.

Overall, during the 4 years EQA time frame, a total of 55 samples, including 15 FASTQ files, three of which synthetic, were provided to the participating laboratories. The four panels included a total of 32 fully susceptible isolates that were correctly characterized by all laboratories; these fully susceptible isolates represented 428 (100%) correct calls.

Single nucleotide polymorphism (SNP) mutations associated with resistance in pure isolates were not always correctly detected: 27 (15%) of 178 resistance-associated calls were missed or incorrectly interpreted (Table 1). In the 2017 panel, an *inhA* c-15t mutation was missed by a single laboratory, while six laboratories failed to report an *embB* M306V mutation. In addition, the panel included a Mycobacterium canettii isolate intrinsically resistant to pyrazinamide due to a M117T mutation in the *panD* gene (15), which was reported by only 3 (23%) of the 13 participating laboratories. In 2018, four laboratories failed to report a *pncA* A46V mutation associated with pyrazinamide resistance, and in 2019, six laboratories did not report a silent *fabG* L203L mutation previously reported to be associated with isoniazid resistance (16, 17).

**TABLE 1:** Summary of the types of determinations scored over the four external quality assessment rounds and the number of errors^*a*^

Determination	Expected no. of calls	Correctly reported	%
No SNPs associated with resistance	428	428	100
Resistance-associated SNPs	166	151	90
SNPs with moderate or weak association with resistance	46	29	63
Deletion/insertion associated with resistance	26	17	65
Resistance-associated SNPs at ≥50% and <90% frequency	34	17	50
Resistance-associated SNPs at ~25% frequency	14	7^*b*^	50
Resistance-associated SNPs at <20% frequency	0	2^*c*^	NA

*^a^*NA, not applicable; SNP, single nucleotide polymorphism.

*^b^*Twelve laboratories identified the mixed genotype but did not detect the resistant loci.

*^c^*Two different laboratories detected 5% L449Q (ctg/cAg) in *rpoB* in two different FASTQ files. These loci were flagged as suspicious and potentially associated with (developing) resistance. These positions were checked by the coordinating laboratory and were found to indeed contain a low number of reads (approximately 3 to 10%) containing this SNP. Their significance is unknown but likely represents low level contamination with DNA from a closely related species.

As expected, mutations associated with an only moderately raised MIC and or a weak association to phenotypic drug resistance (18) were more frequently mischaracterized: 17 (37%) of 46 calls of this type were missed or incorrectly interpreted (Table 2). In 2018, an isolate with a double mutation in the *inhA* gene was included; while the c-15t in the promoter region was correctly identified by all 12 laboratories, the I21T in the coding region was reported by 10 (83%) laboratories. In 2020, a synthetic FASTQ file with mutations associated with an increased MIC to bedaquiline and clofazimine (i.e., position 2 GTG-GCG in Rv0678 loss of start codon [19]) and delamanid (i.e., position 3987106 G > A Trp88Stop in Rv3547 [19]) was included in the panel. Mutations in Rv0678 and Rv3547 were correctly reported by four (33%) and seven (58.3%) laboratories, respectively.

**TABLE 2:** Criteria used for scoring the WGS external quality assessment^*a*^

Error	Points lost
Incorrect drug resistance-associated call (resistance versus susceptibility)	10
Unreported low-confidence resistance mutation or resistance predicted at the incorrect level (high versus intermediate versus low)	5
Failure to report an MDR-TB (yr 2017)^*a*^	25
Failure to detect a mixed genotype	10
Failure to detect rifampicin resistance, 50% or more, in a mixed sample	10
Incorrect genotype (sub/species) assigned to an isolate	10
Incorrect assignment of an isolate to a genetic cluster	6

*^a^*MDR-TB, multidrug-resistant tuberculosis; WGS, whole-genome sequencing.

*^b^*This heavy penalty for missing an MDR-TB was not included in the later panels as more complex isolates mixed with other strains with different sensitivities or less common resistance mutations were included in these panels. Miscalling individual drugs was still penalized with 10 points when a high-confidence mutation was missed.

Of the 26 frame shifts/insertions and deletions associated with resistance, 17 (65.4%) were accurately reported (Table 1). In 2018, a large deletion in the *pncA* gene (223419 to 229307) was included and was detected and correctly interpreted by 7 (58%) of 12 laboratories. In 2019, a single base pair insertion in the *katG* gene was included and correctly detected and interpreted by 10 (71%) of 14 laboratories. In 2020, a deletion in the *embB* gene with unknown significance for the ethambutol MIC and a double single base pair deletion in the *katG* gene were both reported by 4 (50%) of 14 laboratories.

In the 2018 EQA panel, a sample with mixed resistant loci at a 50:50 ratio was correctly detected by 10 (83.3%) of 12 laboratories. Conversely, in the 2019 EQA panel, only 7 (58%) of 12 laboratories correctly reported the resistant mutations of a mixed sample with resistant loci at a 25% frequency, even if the presence of a mixed genotype was reported by the majority (85.7%) of participating laboratories. In 2020, of 11 (78.6%) laboratories able to analyze the 3 synthetic FASTQ files included in the panel, 8 (72.7%) and 7 (63.6%) laboratories, respectively, correctly detected the resistant mutations present at 60% frequency and the *pncA* deletion at 40% frequency (Table 1).

### Identification of lineages.

Identification to the subspecies level was highly accurate in all panels. All laboratories were able to correctly assign the Coll (4) type or genotypic designation to the pure samples included. The naming conventions were consistent within each laboratory but not completely consistent between laboratories. Detection of the mixed genotypes was not required to score full marks; reporting a mixed genotype was sufficient. Nevertheless, in 2019, 9 (64.3%) of 14 laboratories correctly identified the constituents and proportions of the 75:25 mixture of Euro-American (LAM) lineage 4.3.4.2 and East Asian (Beijing) lineage 2.2.1.

### Relatedness analysis.

**FIG 1:**
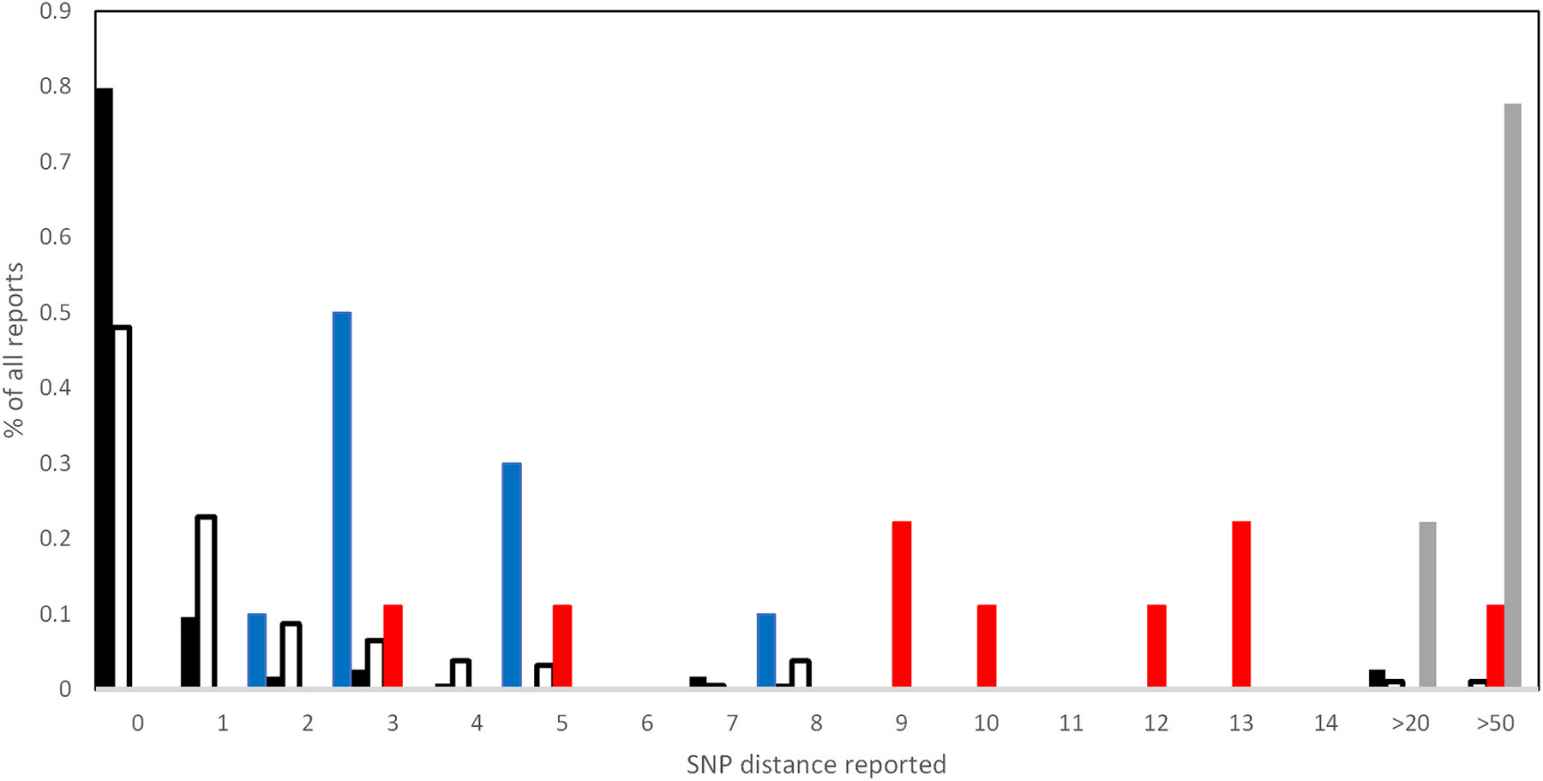
Frequency of scored single nucleotide polymorphism (SNP) distances reported for duplicate samples (black bars), epidemiologically linked samples (empty bars), closely related (approximately 12 SNP distance, red bars), synthetic FASTQ pair (four SNPs and one 3-bp insertion, blue bars), and a genetically close but nonclustered sample (approximately 50-bp distance, gray bars). For the black, empty, and blue bars, a recent epidemiological link should not be ruled out; for the red bars, an epidemiological link is possible but unlikely; and for the gray bars, a recent epidemiological link should be ruled out.

**(i) Duplicate isolates.** Over the 4 years, 10 duplicate isolates were included in the panels. Laboratories were expected to cluster these isolates and indicate they were very closely related, suggesting either possible transmission or laboratory contamination. There were a total of 133 duplicate calls expected, of which 131 (98.5%) were correctly identified as closely related. The single pair of identical isolates missed by one laboratory was due to a clerical error. A precise SNP distance was reported for 114 of these duplicate calls, of which 91 (80%) were reported to have zero SNP distance. The distribution of the SNP distances reported is summarized Fig. 1.

**(ii) Epidemiologically clustered but nonidentical samples.** Over the 4 years, 230 relationships between independent epidemiologically linked isolates were included in the panels. Among those, 220 (95.7%) were correctly reported by the laboratories as potentially epidemiologically linked. The 10 missed links were due to a failure to correctly cluster a DNA sample with a FASTQ file. For 170 (77.3%) of the identified links, a precise SNP distance was provided, including three links reported as likely to be epidemiologically linked but with a SNPs distance of 20 (Fig. 1). In 2019, a sample closely related to two other clustered isolates was included in the panel with an estimated SNP distance of 12 SNPs (based on the organizing laboratories analytical pipeline). The SNP distances reported for this sample are indicated in Fig. 1.

Two synthetic FASTQ files in the 2020 panel differed by three SNPs. The two samples were correctly identified by all laboratories as clustered, and 10 (71%) laboratories reported an exact SNPs distance between these isolates which ranged from 1 to 7 SNPs (blue bars in fig. 1).

Overall, there were 18 incorrect epidemiologically linked samples reported; 1 was the result of a clerical error already mentioned above, while the remaining 17 were the result of unrelated isolates being incorrectly joined by the clustering algorithm. This was due to the presence of a DNA sample containing a mixture of two isolates belonging to different clusters in both the 2018 and 2019 panels. In 2017 and 2020, a genetically related but not clustered sample with an approximate distance of 50 SNPs was included in the panels. In 2017, none of the 12 laboratories considered this sample epidemiologically linked with any other sample in the panel, while in 2020, one of 14 laboratories indicated this sample as closely related to a cluster. The laboratories that reported an SNP distance for these pairs estimated a distance of between 23 and 81 SNPs, with an average of 64 SNPs (gray bars in Fig. 1).

## DISCUSSION

Of the 32 EU/EEA TB NRLs invited to participate, almost 50% (between 12 and 15) joined this initiative. They represent the current EU/EEA laboratories routinely using WGS or in the process of implementing this technology to characterize M. tuberculosis isolates. A rapid switch to the use of M. tuberculosis WGS is under way, but as this methodology is not yet fully standardized, the development of an external quality assurance system is critical to assess and compare laboratories’ proficiency. M. tuberculosis WGS-based genotyping has been shown to be superior to MIRU-VNTR-based genotyping with respect to resolution, reproducibility, and cluster detection (8, 12, 13), and the use of WGS for prediction of resistance and susceptibility to anti-TB drugs shows great promise, even if the precise resistance mechanisms for new and repurposed drugs remain uncertain. In addition, laboratories value M. tuberculosis WGS as a potential cost-saving opportunity, for instance by reducing the need for phenotypic drug susceptibility testing (DST) in the presence of fully susceptible isolates.

Importantly, the recent publication by the World Health Organization (WHO) of a curated list of M. tuberculosis mutations and their associated link with resistance/susceptibility provides a basis for future development of M. tuberculosis WGS, including its use as the reference method for specific drugs and EQAs. This publication represents a significant development with respect to the standardization and acceptance of this methodology for M. tuberculosis complex drug susceptibility prediction (20).

### Genotyping.

Overall, the results of WGS-based mycobacterial genotyping were more accurate compared to the ones previously observed in EQA rounds based on MIRU-VNTR typing (12). Nonetheless, some laboratories did report structurally high SNP distances for closely related isolates, even if the isolates were correctly reported as potentially related. This is likely due to incomplete filtering of noise in the SNP-calling algorithm, often caused by not excluding all poorly mapped reads, used by different laboratories, which makes the comparison of precise SNP distances provided by the EQA participants potentially misleading (21).

The presence of SNP-calling noise was further confirmed by the analysis of the duplicate isolates included in the EQA panel. In this case, only 67% of the links were identified with a zero SNP distance (Table 1; Fig. 1). Thus, low numbers of SNPs should be treated with caution when investigating transmission chains, as in many pipelines, a proportion of these SNPs may be due to artifacts (Fig. 1). In addition, the inclusion of two synthetic FASTQ files generated from an edited reference sequence (22) and differing by three single SNPs, one double nucleotide change, and one 3-bp insertion (sample 12 and 13 in Table 3) were reported by participating laboratories as differing by a number of SNPs ranging from one to seven, which is in line with previous findings (21). These minor differences between pipelines, although having only minor effects when detecting potentially epidemiologically linked isolates, may have implications when inferring transmission chains as the natural accumulation of mutations in M. tuberculosis is extremely slow (9).

**TABLE 3:** Composition and scored characteristics of the external quality assessment panels^*a*^

Year	Sample	Links scored with other samples from the same yr			Coll identity (4)	Expected susceptibility	Resistance-associated mutations scored							
		Identical to	Clusters with	>12 bp, <100 bp			INH	RIF	EMB	ETH	PZA	STR	FLQ	Other resistance
2017	DNA1		3, 5	7, 9	3.1.1	Susceptible first line								
2017	DNA2	6			4.4.1.1	Susceptible first line								
2017	DNA3	5	1	7, 9	3.1.1	Susceptible first line								
2017	DNA4				M. canettii	Mono-resistant					panD M117T (atg/aCg)			
2017	DNA5	3	1	7, 9	3.1.1	Susceptible first line								
2017	DNA6	2			4.4.1.1	Susceptible first line								
2017	DNA7	9		1	3.1.1	Susceptible first line								
2017	DNA8				2.2.1	MDR	InhA c-15t	rpoB S450L (tcg/tTg)	embB E405D (gag/gaC)	inhA c-15t		rpsL_K43R (aag/aGg)		
2017	DNA9	7		1	3.1.1	Susceptible first line								
2017	DNA10				4.9	Susceptible first line								
2018	DNA1		6, 14		4.3.3	Susceptible first line								
2018	DNA2				2.2.1	MDR	katG S315T (agc/aCc)	rpoB S450L (tcg/tTg)	embB M306V (atg/Gtg)		pncA deleted (223419 to 229307)			
2018	DNA3		5, 9, 11		3	Susceptible first line								
2018	DNA4		8, 10		4.1.2.1	Susceptible first line								
2018	DNA5	9	3, 11		3	Susceptible first line								
2018	DNA6		1, 14		4.3.3	Susceptible first line								
2018	DNA7				4.1.2.1 + 2.2.1 (Mix 8 + 10)	Mono-resistant (mixed 50% resistant)		rpoB H445Y (cac/Tac)						
2018	DNA8	10	4		4.1.2.1	Mono-resistant		rpoB H445Y (cac/Tac)						
2018	DNA9	5	3, 11		3	Susceptible first line								
2018	DNA10	8	4		4.1.2.1	Mono-resistant		rpoB H445Y (cac/Tac)						
2018	FASTQ11		3, 5, 9		3	Susceptible first line								
2018	FASTQ12		13		4.8	Susceptible first line								
2018	FASTQ13		12		4.8	Susceptible first line								
2018	FASTQ14		1, 6		4.3.3	Susceptible first line								
2018	FASTQ15				2.2.1	MDR	inhA c-15t + I21T (atc/aCc)	rpoB S450L (tcg/tTg)	M306V (atg/Gtg)	inhA c-15t	pncA A46V (gca/gTa)			
2019	DNA1	10	13, 15		3.1.1	Susceptible first line								
2019	DNA2				1.1.2	Mono-resistant					panD I49V (atc/Gtc, novel)			
2019	DNA3				2.2.1 + 4.3.4.2 (Mix 9 + 4)	MDR (mixed 20% res)	katG insertion (2156066 T)	rpoB S450L (tcg/tTg)				rpsL K43R (aag/aGg)	gyrA D94G (gac/gGc)	
2019	DNA4	6	6, 7, 12		4.3.4.2	Susceptible first line								
2019	DNA5				M. caprae	Susceptible first line								
2019	DNA6	4	6, 7, 12		4.3.4.2	Susceptible first line								
2019	DNA7		4, 6, 12		4.3.4.2	Susceptible first line								
2019	DNA8				3	Mono-resistant							gyrA A90V (gcg/gTg)	
2019	DNA9				2.2.1	MDR	katG insertion (2156066 T)	rpoB S450L (tcg/tTg)				rpsL K43R (aag/aGg)	gyrA D94G (gac/gGc)	
2019	DNA10	1	13, 15		3.1.1	Susceptible first line								
2019	FASTQ11				M. avium + M. tuberculosis	NA								
2019	FASTQ12		4, 6, 7		4.3.4.2	Susceptible first line								
2019	FASTQ13		1, 10, 15		3.1.1	Susceptible first line								
2019	FASTQ14				4.8	Mono-resistant		fabG1 L203L (ctg/ctA, silent)						
2019	FASTQ15		1, 10, 13		3.1.1	Susceptible first line								
2020	DNA1	15	8, 9, 14	7	4.8	Susceptible first line								
2020	DNA2				4.2.2.1	MDR	katG S315T (agc/aCc)	rpoB L452P (ctg/cCg)	embB G497R (cag/cGg), embA_Cc-16t		pncA V7G (gtc/gGc)	rpsL K88R (aag/aGg)	gyrA D94A (gac/gCc)	Low-confidence BDQ/CFZ Rv0678 L74M (ctg/Atg), low-confidence
2020	DNA3				4.1.2.1	Susceptible first line (Rv0678 mutation, low-confidence)								Rv0678 D5G (gac/gGc), low-confidence BDQ
2020	DNA4				BOV	Mono-resistant					pncA H57D (cac/Gac)			
2020	DNA5				3	RIF (low-confidence EMB)		rpoB S450L (tcg/tTg)	embB deletion (4246528-4246587)					
2020	DNA6	10			3	Resistance uncertain (INH double mutation)	katG deletion (2155059 1 bp) + insertion (2155065 1 bp)							
2020	DNA7			1, 8, 9, 14, 15	4.8	Susceptible first line								
2020	DNA8	14	1, 9, 15	7	4.8	Susceptible first line								
2020	DNA9		1, 8, 14, 15	7	4.8	Susceptible first line								
2020	DNA10	6			3	Resistance uncertain (INH double mutation)	katG deletion (2155059 1 bp) + insertion (2155065 1 bp)							
2020	SyFASTQ11		12, 13		4.1.1.3	MDR (mixed 40% to 60%)	katG S315T (agc/aCc)	rpoB S450L (tcg/tTg) 60%			pncA del2289219 to 2289232 40%			
2020	SyFASTQ12		11, 13		4.1.1.3	RIF, INH, PZA	katG S315T (agc/aCc)	rpoB S450L (tcg/tTg)			pncA del2289219 to 2289232			
2020	SyFASTQ13		11, 12		4.1.1.3	Pre-XDR	katG S315T (agc/aCc)	rpoB S450L (tcg/tTg)			pncA del2289219 to 2289232			Low-confidence BDQ/CFZ Rv0678 V1A (gtg/gCg), DLM ddn Trp88_W88* (tgg/tAg), low-confidence
2020	FASTQ14	8	1, 9, 15	7	4.8	Susceptible first line								
2020	FASTQ15	1	8, 9, 14	7	4.8	Susceptible first line								

*^a^*BDQ, bedaquiline; CFZ, clofazimine; EMB, ethambutol; FLQ, fluoroquinolone; INH, isoniazid; MDR, multidrug-resistant; PZA, pyrazinamide; RIF, rifampicin; STR, short tandem repeat; XDR, extensively drug-resistant.

The inclusion in the panel of samples containing a mixed genotype between isolates belonging to two different clusters resulted in over clustering when the analysis was performed by core genome multilocus sequence typing (cg-MLST). In this case, two unrelated clusters were combined in one cluster with the mixed genotypes isolates linking the two unrelated clusters together. This was largely a consequence of lack of familiarity with the characteristics of the specific clustering algorithms used, as in all cases the laboratories correctly identified the different genotypes (Coll clades) of the artificially clustered isolates. The naming conventions were consistent within each laboratory but not completely consistent between laboratories; this problem related to the complex publication history of these names and was not encountered for the Coll system (4). For this reason, for communication between laboratories, the Coll classification system is favored and will be required in future rounds of this EQA.

Given the lack of a standardization between different analytical pipelines, the interlaboratory comparison of WGS genotypic results is not fully accurate. Therefore, the primary way to check whether a cluster identified by one laboratory is linked to a cluster identified by another laboratory is by sharing strains, DNA, or FASTQ files between the two sites and having them analyzed by a single pipeline.

This is a critical issue that was partially addressed within the EuSeqMyTB initiative (14, 23) by generating a central WGS data repository in which all sequence data of rifampicin-resistant/multidrug-resistant M. tuberculosis strains were analyzed using a common pipeline. Importantly, the lessons learned from this pilot initiative served as a base for the development by the European Centre for Disease Prevention and Control (ECDC) of a WGS-based surveillance system for M. tuberculosis within the newly established EpiPulse platform (https://www.ecdc.europa.eu/en/news-events/launch-epipulse-new-portal-strengthen-prevention-and-control-infectious-diseases).

### Prediction of drug susceptibility.

The use of WGS for the prediction of drug susceptibility was highly accurate, as all laboratories over the 4 years of this study correctly identified all fully susceptible strains. Similarly, laboratories showed good performance in the detection of mutations associated with drug resistance. It should be noted that that these EQA rounds were performed prior to the publication of the WHO catalogue (20). The most common errors were related to: (i) intrinsic limitations of the analytical pipeline used (e.g., unable to detect large gene deletions); (ii) interpretation and reporting of specific resistance-associated mutations present in genes not routinely screened by laboratories (e.g., mutation M117T in the *pan*D gene conferring PZA resistance); and (iii) clerical errors. The second type of error is likely overemphasized in this exercise, as in order to challenge laboratories, rarely encountered mutations were included, and during this exercise, no standard curated list of potentially resistance-associated was available.

All mutations with strong association to phenotypic drug resistance were expected to be reported and the impact on the associated phenotypic resistance was expected to be indicated. Mutations with a moderate or weak association to phenotypic drug resistance were expected to be identified, and further investigations, for example by phenotypic testing, were expected to be suggested. In 2017, one laboratory failed to report a high-confidence mutation for isoniazid resistance located in the gene upstream region. In this case, the specific pipeline used correctly detected the mutation, but it was, however, missed by the user because only the gene-coding regions were included the screening scheme.

Mutations in genes of interest with unknown or weak association with resistance were obviously more challenging (18). The *fabG1* L203L mutation (16, 17) included in 2019 was detected and reported as significant by 8 of 14 laboratories but correctly linked to isoniazid (INH) resistance by only 7 laboratories. When this omission was communicated, one laboratory, which missed the phenotype associated with this mutation, indicated that they have previously seen this mutation but did not link it with an INH-resistant phenotype. This raises an interesting reporting problem, as three laboratories indicated they would not report an undocumented mutation with unknown phenotypic implications to their clinical colleagues. Reporting standards are an ongoing discussion and future EQA panels should reflect these discussions. The recently published list of mutations by the WHO addresses this critical issue (20) and will be used to inform the scoring of this EQA in the following rounds. Nonetheless, even before this list became available, many laboratories were able to identify and even interpret novel mutations. For example, the single base pair deletion/insertion in samples 6 and 10 in the 2020 panel was reported, with 50% of laboratories suggesting confirmation of the resistant phenotype by MIC determination and drug susceptibility testing. This emphasizes the great potential of WGS for MIC prediction as the knowledge base builds (24).

In conclusion, this EQA activity was designed to assess and challenge M. tuberculosis WGS pipeline results, interpretation, and reporting—and we believe helped improve—the quality of M. tuberculosis WGS analysis in the EU/EEA particularly with respect to the prediction of drug resistance/susceptibility. Intralaboratory genotyping capacity was also assessed, and with the use of the shared FASTQ files, an assessment of interlaboratory reproducibility was also attempted. Overall, WGS-based genotyping EQA results were more reliable and accurate than those based on the previously used reference method, MIRU-VNTR. Even if for now we limited the evaluation to the laboratory capacity to identify likely related strains rather than directly comparing SNPs distance between isolates, we believe that future EQA rounds should look more carefully at the different performance of individual pipelines and help identifying criteria for standardization.

### Study limitations.

We recognize that the WGS EQA scheme described in this paper has some limitations. We assessed the laboratories’ capacity to analyze WGS data with respect to drug resistance prediction and relatedness analysis but have not directly evaluated the quality of the generated raw sequence data nor compared the different analytical pipelines or the performance of the various sequencing technologies used (i.e., Illumina platforms and IonTorrent). Although only one laboratory used the IonTorrent platform, we did not observe any impact on the laboratory capacity to detect the resistance-associated mutations or on its capacity to identify likely related clones.

Although the quality of the raw sequence data generated by the different laboratories was not assessed, we did include in the EQA panel five FASTQ files, which would have indirectly indicated if the quality of the raw sequence data was the primary source of errors. This did not appear to be the case. However, the inclusion of these files did highlight some difficulties of some participants to effectively cluster genetically closely related isolates when the WGS sequence was not generated locally. This was likely due to inexperience or possibly by the lack of standardized protocols for the processing and downstream analysis of the shared FASTQ files compared to the ones generated locally. If this is indeed the case, we expect it to be resolved in future EQA rounds.

Second, during the study period (2017 to 2020), there was no single globally endorsed list of resistance-associated mutations for M. tuberculosis complex to guide data interpretation. However, the recent publication by WHO of the catalogue of mutations in M. tuberculosis complex and their association with drug resistance (20) resolves this limitation and provides a standard to be used in future EQA rounds.

Third, although more rapid (or slightly delayed) reporting of EQA results was not scored, we acknowledge the importance of providing WGS results within short turnaround times. Therefore, we will discuss the possibility of scoring this indicator in future EQA schemes.

In addition, given the increasing relevance of nontuberculous mycobacteria (NTMs) infections within our network and globally, we will consider the inclusion of more NTM species within future EQA panels. Finally, we acknowledge that fact that this EQA activity is not ISO accredited.

## MATERIALS AND METHODS

### Participants.

National TB reference laboratories in the EU/EEA routinely performing WGS genotyping of M. tuberculosis isolates or in the process of establishing this service were invited to enroll in the EQA scheme for WGS. A total of 13, 12, 14, and 15 laboratories within the ERLTB-Net-2 participated in 2017, 2018, 2019, and 2020, respectively. The National Institute for Public Health and the Environment (RIVM) coordinated test panels preparation, distribution, data analysis, and laboratory certification (Fig. S1).

### EQA panel composition.

The four test panels prepared in 2017, 2018, 2019, and 2020 consisted of ten high molecular weight genomic DNA from mycobacterial isolates, supplemented, in year 2018 onwards, with five FASTQ files generated in the organizing laboratory. Then, in 2020, three FASTQ files were replaced by synthetic files to allow for additional flexibility with the inclusion of specific variants not represented in the isolate collection of the organizing institution (Table 3). The panels prepared at the RIVM were distributed as noninfectious inactivated DNA to participating laboratories. The laboratories were asked to perform WGS typing using their standard methodology and to report the results to the RIVM.

Reference results were based on the in-house analysis of WGS sequence data obtained in the RIVM (10) supplemented with phenotypic-based DST data routinely obtained from the selected isolates. As the majority of M. tuberculosis isolates in the EU/EEA are phenotypically sensitive to first line antimycobacterial drugs, all panels included at least 50% sensitive isolates, providing the possibility to assess the specificity of drug resistance prediction (i.e., detection of false-positive resistance calls). The remaining M. tuberculosis strains were mono- or multidrug-resistant isolates carrying a mix of frequently encountered and rare mutations associated/interim-associated with drug resistance. Strains with rarely encountered mutations in genes associated with drug resistance but with uncertain or no documented association with drug resistance were also included.

All panels contained either two or three duplicate samples consisting of exactly the same DNA. In 2018 and 2019, duplicates consisted of extracted DNA and data obtained by the organizing laboratory shared in the form of FASTQ files. Genetically close isolates (≥12 to 50 SNPs apart) not considered to be part of a recent cluster, according to the RIVM pipeline (10), were also included in two panels.

The panels also included DNA from different members of the M. tuberculosis complex, as well as other mycobacterial species that were expected to be identified as nontuberculous isolates. The laboratories were asked to define the bacteria at the species/subspecies level.

Finally, panels included artificial mixtures of DNA from two different M. tuberculosis complex strains at specific ratio to challenge the laboratories WGS analysis pipeline by mimicking mixed infections or potential laboratory cross-contaminations. The composition of the different EQA panels from 2017 to 2020 is outlined in Table 3.

In 2020, three synthetic FASTQ files (200-bp reads) were included in the panel. They were generated from a reference M. tuberculosis reference FASTA (AE000516.2 CDC1551 complete genome [25]) file by CuReSim, a customized tool using the default options to generate synthetic new-generation sequencing reads (22). Mutations were manually introduced into the reference sequence and mixed alleles created by combining synthetic reads generated from different input files by CuReSim and combining them into a single file in the desired ratio. Table 4 reports the specific mutations introduced into the three synthetic FASTQ file in 2020 and their respective frequency. Of note, the results from the analysis of the synthetic FASTQ files did not contribute to the overall EQA assessment score due to their novelty but still reported to the participating laboratories for information.

**TABLE 4:** Mutations introduced into genome sequence AE000516.2 CDC1551 in the synthetic FASTQ prepared for the 2020 panel

Year	Sample	Gene	Mutation introduced (position in reference genome)	Frequency (%)	Interpretation
2020	11	*rpoB*	Ser450Leu C > T (761155)	60	Rifampicin-resistant
		*katG*	Ser315Thr G > C (2155168)	60	Isoniazid-resistant
		*pncA*	14-bp deletion (2289219 to 2289232)	40	Pyrazinamide-resistant
		*nirB*	Lys686Lys A > G (304923)	100	NA
2020	12	*rpoB*	Ser450Leu C > T (761155)	100	Rifampicin-resistant
		*katG*	Ser315Thr G > C (2155168)	100	Isoniazid-resistant
		*pncA*	14-bp deletion (2289219 to 2289232)	100	Pyrazinamide-resistant
		*nirB*	Lys686Asn A > G>C (304923)	100	NA
2020	13	*rpoB*	Ser450Leu C > T (761155)	100	Rifampicin-resistant
		*katG*	Ser315Thr G > C (2155168)	100	Isoniazid-resistant
		*pncA*	14-bp deletion (2289219 to 2289232)	100	Pyrazinamide-resistant
		*ddn* (Rv3547)	Trp88Stop G > A (3987106)	100	Possible delamanid resistance (27)
		*Rv0678*	Met1Ala T > C (778991), loss of start codon	100	Possible bedaquiline resistance (19)
		*nirB*	Lys686Asn A > G>C (304923)	100	NA
		*Rv1985C*	Pro34Pro G > C (2229801)	100	NA
		*Rv2845c (lipQ)*	Arg60Leu C > A (2793810)	100	NA
		*glpK*	Insertion CCC (4139190)	100	NA
		*Rv1592c*	Ile322Leu T > G (1792777) and Glu321Asp T > A (1792778)	100	NA

### EQA panel preparation.

DNA was extracted using the CTAB method (26). The DNA was prepared exactly as previously described for MIRU-VNTR EQA panels (12). Briefly, DNA concentration was measured with the Nanodrop ND-1000 system (Thermo Scientific, Wilmington, DE). For the preparation of test panels, the DNA samples were diluted to a final concentration of 100 ng/μL. The test panels were stored at 4°C and shipped to registered laboratories at room temperature by a courier service. FASTQ files were made available for download from a link provided by email to laboratories who agreed to participate.

### EQA results assessment and reporting.

Laboratories were expected to report the methodology used, any mutations regarded as significant with respect to drug resistance, and the genotype of the isolates, as well as to indicate isolates they regarded as genetically closely related (potentially clustered). Finally, laboratories were requested to provide an interpretation of these results based on their standard reporting of WGS sequence results for routine isolates. Duplicates and isolates belonging to a molecular cluster, defined by a six SNPs distance threshold, were correctly scored if reported as closely related. The reporting of the exact genetic SNPs distance between the different isolates was not mandatory but recorded when provided. The RIVM compared the results from each laboratory to those expected and reported the individual results to each of the laboratories. Laboratories were given the option to query any discrepancies.

An initial conclusion on the submitted EQA results was communicated to each laboratory. Participants were able to query specific results or ask for clarifications to the organizing laboratory. Points were deducted if isolates were not correctly identified or if drug resistance-associated mutations were not reported or correctly interpreted. The criteria used for scoring the EQA results slightly varied over the years (Table 2) to reflect the changes of the EQA panel composition. An EQA WGS proficiency certificate was granted to laboratories that scored at least 75 of 100 points.

### Data availability.

All WGS data generated at RIVM for this study are deposited as FASTQ files in the Sequence Read Archive of the National Center for Biotechnology Information (accession number PRJNA896516).
