# Supplementary material for: Experiences from 4 Years of Organization of an External Quality Assessment for Mycobacterium tuberculosis Whole-Genome Sequencing in the European Union/European Economic Area
Source: Microbiol Spectr. 2022 Dec 8;11(1):e02244-22. doi: 10.1128/spectrum.02244-22 (PMC9927412; doi:10.1128/spectrum.02244-22)
Supplement: Supplemental file 1 — Fig. S1. Download spectrum.02244-22-s0001.pdf, PDF file, 0.10 MB [file spectrum.02244-22-s0001.pdf]

Supplementary Figure 1. EQA Workflow

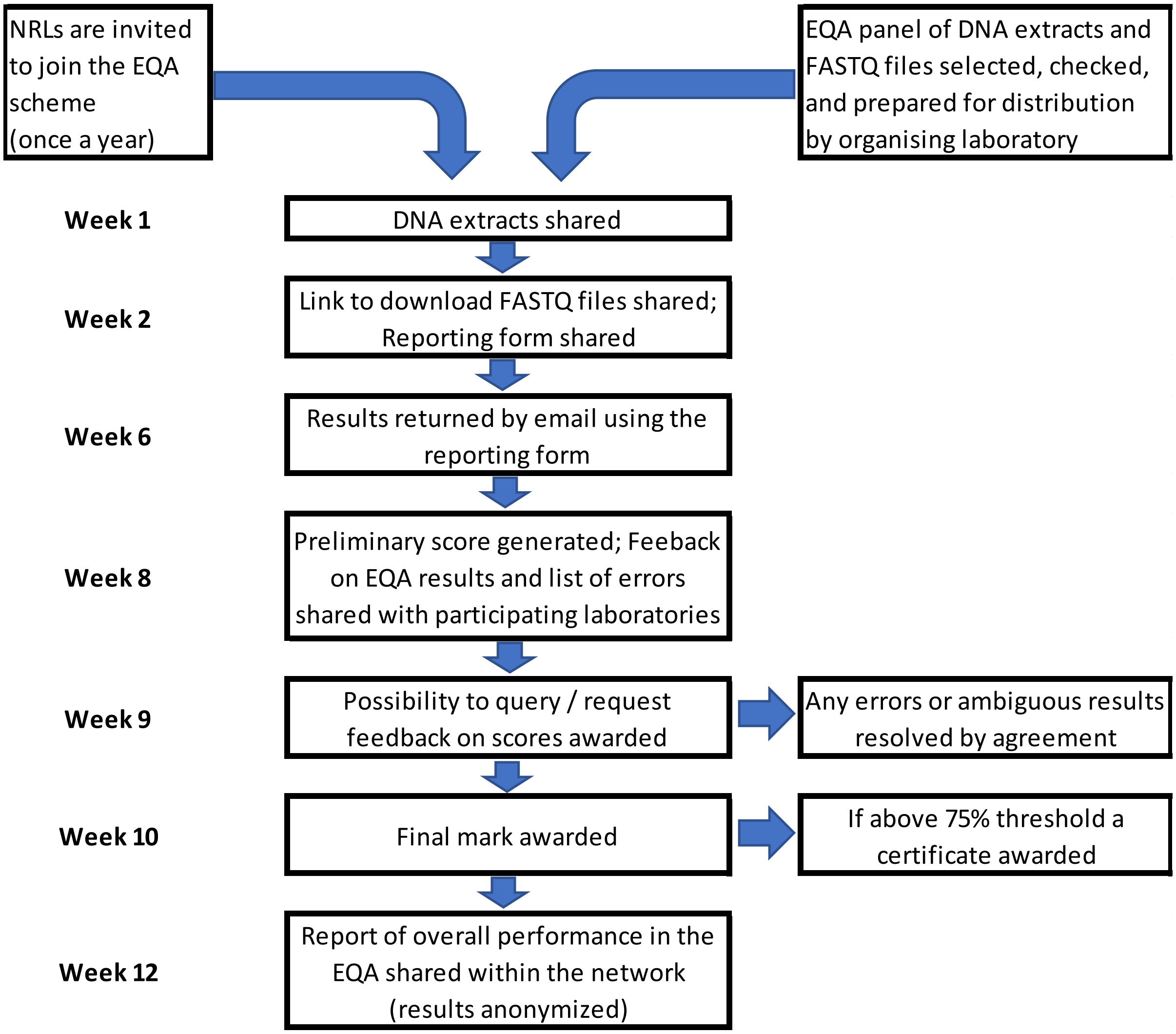

NRL = EU/EEU National TB reference laboratories
EQA = External quality assessment
